# Multiple Nabothian Cysts and Tunnel Clusters Clinically Mimicking Lobular Endocervical Glandular Hyperplasia: A Case Report

**DOI:** 10.7759/cureus.77643

**Published:** 2025-01-18

**Authors:** Kenji Yorita, Misaki Murayama, Kimiko Nakatani, Miho Tsutsui, Hiroshi Yoshida

**Affiliations:** 1 Diagnostic Pathology, Japanese Red Cross Kochi Hospital, Kochi, JPN; 2 Obstetrics and Gynaecology, Japanese Red Cross Kochi Hospital, Kochi, JPN; 3 Radiology, Japanese Red Cross Kochi Hospital, Kochi, JPN; 4 Diagnostic Pathology, Kochi University Hospital, Nankoku, JPN; 5 Diagnostic Pathology, National Cancer Center Hospital, Chuo, JPN

**Keywords:** gastric-type adenocarcinoma, lobular endocervical glandular hyperplasia, nabothian cyst, tunnel cluster, uterine cervix

## Abstract

This report describes a 48-year-old woman who presented with multiple cystic lesions in the uterine cervix. Magnetic resonance imaging (MRI) showed multiple large and small cysts in the uterine cervix, which raised lobular endocervical glandular hyperplasia (LEGH) as a differential diagnosis, because a cosmotic and microcystic pattern characteristic of LEGH might be suggested. However, pathological examination of the conization specimen revealed the simultaneous occurrence of superficial and deep Nabothian cysts (NCs) and tunnel clusters (TCs), without evidence of LEGH. LEGH is a potential precursor lesion to gastric-type adenocarcinoma (GAC) and should be distinguished from NCs and TCs, which do not typically require follow-up. This case highlights the challenges in the clinicoradiological differentiation between benign cervical lesions and LEGH. Clinicians and pathologists should be aware of the possibility of coexisting NCs and TCs mimicking LEGH in imaging studies.

## Introduction

Common cystic lesions in the cervix are Nabothian cysts (NCs), tunnel clusters (TCs), lobular endocervical glandular hyperplasia (LEGH), and gastric-type adenocarcinoma (GAC), including minimal deviation adenocarcinoma [1,2]. NCs, TCs, and LEGH are benign, whereas GAC is malignant. Although it is important to distinguish malignant diseases, it is also important to distinguish LEGH from NCs and TCs, because LEGH can be a precursor lesion of GAC [3,4], whereas NCs and TCs are benign diseases and are not considered precursor lesions of cancer [1,5]. Magnetic resonance imaging (MRI) is known to be an effective modality for differentiating NC, LEGH, and GAC. NC can be suspected in coarse, multiple large cystic lesions; LEGH, when there is a central solid lesion or multiple small cysts surrounded by large cysts (cosmos pattern), or when there is an aggregation of small cysts without peripheral large cysts (microcystic pattern); and GAC, when the solid component is mainly seen [1,4]. In addition, cosmos patterns in MRI images have also been reported as suspicious findings for gastric-type mucin-positive cystic lesions of the cervix, including GAC and LEGH [6]. Regarding the imaging findings of TCs, no coherent case studies have been conducted, probably because TCs, which have two histological types - non-cystic (type A) and cystic (type B) - are often discovered incidentally [5,7].

Here, we report the case of a 48-year-old woman with multiple cystic lesions in the cervix, which had been detected due to lower abdominal pain over the past six months. Although LEGH was suspected on MRI, the pathological diagnosis of a cervical conization specimen revealed multiple coexisting NCs and TCs without LEGH. Such cases are rare, with only one reported case in the PubMed database [1]. Clinicians and pathologists should be aware of this benign case, radiologically mimicking LEGH.

## Case presentation

**Figure 1:**
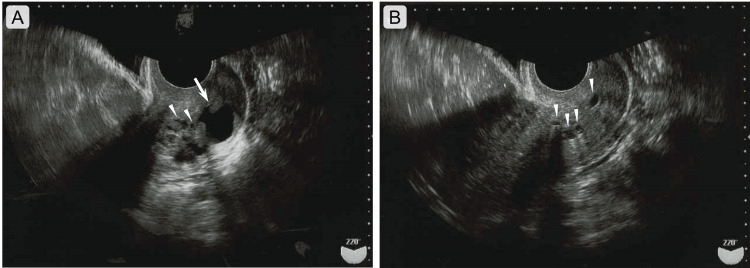
Transvaginal ultrasonographic images of multiple Nabothian cysts and tunnel clusters in the uterine cervix. Large, small, low-echoic lesions, suggestive of various-sized cysts, are present in the uterine cervix. The large cyst is indicated by an arrow in (A). The small cystic lesions are shown using arrowheads in (A)-(B). The scale bars indicate photographs.

A 48-year-old woman, para 2, gravida 2, noticed lower abdominal pain six months ago and visited a nearby hospital. Both births were vaginal deliveries. There was no significant medical or family history. Vital signs were unremarkable. Physical signs suggestive of Peutz-Jeghers syndrome were absent. There was no irregular vaginal bleeding, and the discharge was clear, never excessive or increased. There was no pain or bleeding during sexual intercourse. She did not appear to be doing anything special about contraception. Complete blood and serological test results were unremarkable. Transvaginal ultrasonography revealed small and large cystic lesions in the cervix, and no abnormalities in the bilateral ovaries. No abnormal findings were observed on colposcopy, and cytological examination of the uterine cervix revealed no atypical cells. Although the cause of the abdominal pain was unknown, the patient was referred to our hospital for evaluation of the cervical lesions. At our hospital, transvaginal ultrasonography also confirmed multiple cysts of various sizes in the uterine cervix, with a maximum size of 16 mm (Figure 1).

**Figure 2:**
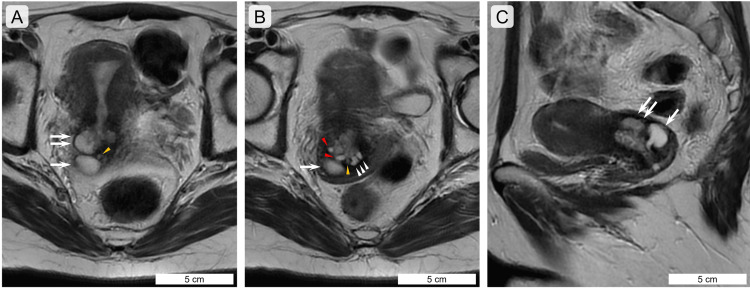
Magnetic resonance imaging photos of multiple Nabothian cysts and tunnel clusters in the uterine cervix. (A)-(C) T2-weighted images (A-B, axial sections; C, sagittal section) of magnetic resonance imaging (MRI) of the uterine cervical lesions. Images (A) and (B) are serial axial sections, and the photograph of (B) shows a more foot-directed section of the photograph in (A). Large, small, high-intensity, and demarcated lesions suggesting cysts are seen in the uterine cervix. In comparison with pathological studies, the two deep Nabothian cysts (NCs) are indicated by arrows and double arrows, and the superficial NCs are indicated by red arrowheads. Most tunnel clusters (TCs) cannot be identified using MRI; however, TCs may be indicated by orange arrowheads as lower-intensity lesions. Two superficial NCs (indicated by red arrowheads) are present on the luminal side of the deep NCs. The scale bars indicate photographs.

MRI also confirmed cysts of various sizes, from the inner to the outer halves of the cervical wall (Figures 2A-2C), without abnormal findings in the uterine body and bilateral adnexa. Two large cystic lesions reached the periphery of the uterine wall (Figures 2A, 2C). Smaller cystic lesions were also observed; two small cystic lesions were found on the luminal side of the cervix, near the two large cystic lesions (Figures 2A, 2B). Solid and/or invasive portions were not found, and contrast-enhanced lesions were not observed. Cosmotic and microcystic patterns observed on MRI could not be ruled out. These patterns are MRI findings suggestive of LEGH [1,4]; therefore, LEGH was included in the differential diagnosis.

We opted for conization for diagnostic purposes. Two large cystic lesions could be partially sampled. There were no problems during the surgery, postoperative bleeding was minimal, and the patient was discharged the day after surgery.

**Figure 3:**
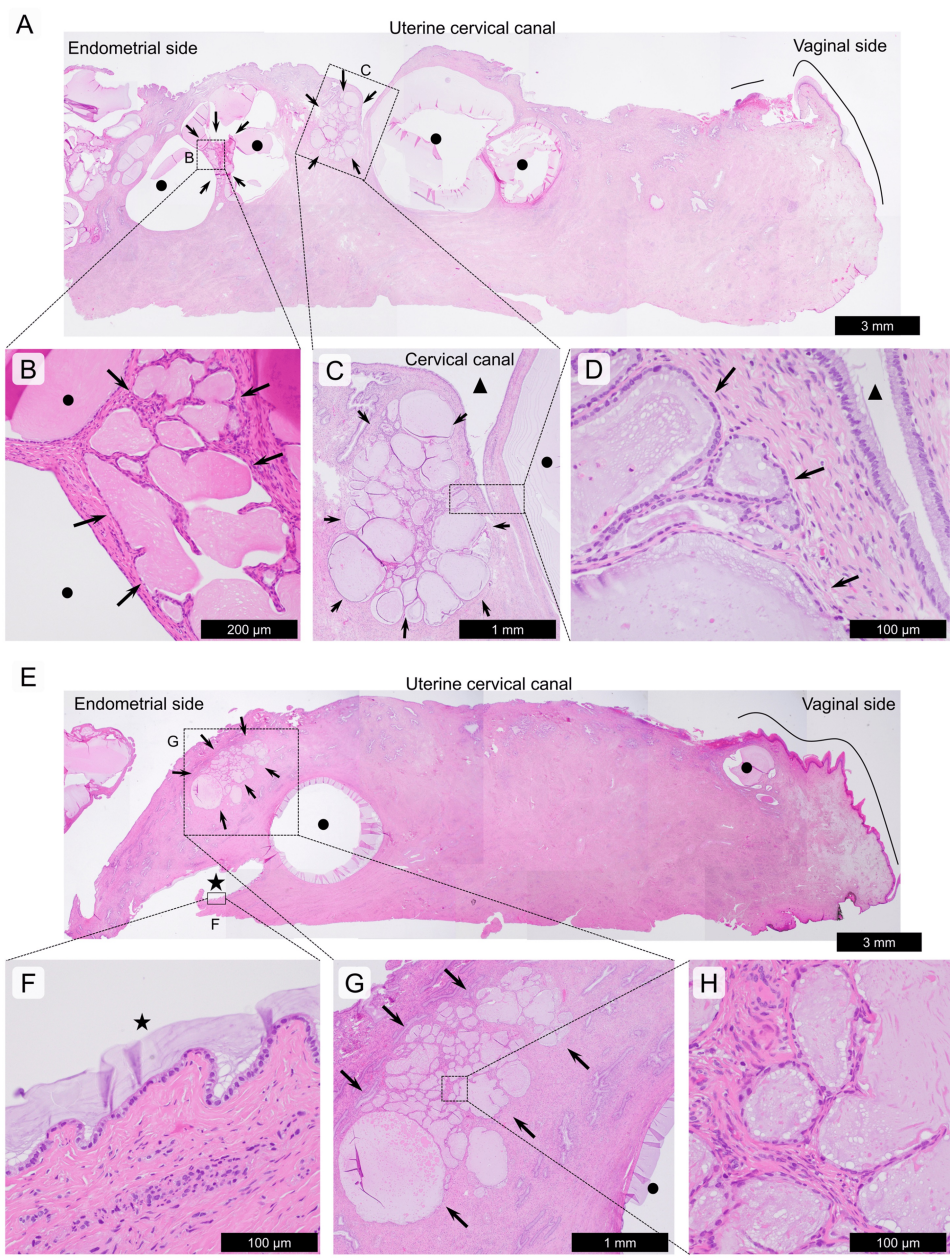
Histological features of multiple Nabothian cysts and tunnel clusters in the uterine cervix. (A)-(D): Multiple superficial Nabothian cysts (NCs) and tunnel clusters (TCs). A low-magnification photo of the 4 o’clock section of the conization tissue is shown in photo (A). Multiple superficial NCs (dots indicate cystic spaces of superficial NCs) and multiple cystic types of TCs (indicated by arrows) are closely and intimately present on the luminal side of the uterine cervix. Photo (B) shows the cystic lesion centrally, including the TC, and the peripheral localization of the NCs. No atypia is seen in the epithelium of NCs and TCs. Photos (B) and (C) are magnified images of the (B) and (C) squares in (A), respectively. Photo (D) is a magnified image of the square in photo (C). (E)-(H): Deep NC and TC. A low-magnification photograph at the 7 o’clock position of the conization tissue is shown in photo (E). The deep NC (stars indicate cystic spaces of deep NCs), exposed at the vertical margin, and a smaller NC (dots indicate cystic spaces of superficial NCs) are closely present on the luminal side of the deep NC in photo (E). The low cuboidal epithelium of the deep NC shows no atypia in photo (F). A cystic TC is present above the deep NC (G)-(H). Photos (F) and (G) are magnified images of squares (F) and (G) in (E), respectively. Photo (H) is a magnified image of the square in (G). Black lines in (A) and (E) indicate localization of the stratified squamous epithelium. Triangles indicate uterine cervical canal spaces. The scale bars indicate photographs.

The conization specimen was divided into 12 sections, all of which were histologically evaluated. From the 3 to 10 o’clock positions, we found multiple small and large cysts, and multiple well-demarcated microscopic nodules with clusters of dilated glandular ducts (Figure 3). The epithelium of the cysts and glands was a flat to low cuboidal monolayer, with areas of mild swelling over the nuclei of the background cervical gland epithelium; however, there was no mitosis, papillary growth on the luminal side, or infiltrative growth with stromal reaction. Immunostaining (MUC6, HIK1083, claudin 18, and carbonic anhydrase IX) was performed to investigate gastric-type immunophenotype. MUC6 was partially positive in some NCs and TCs, but the other three markers were negative. The epithelium was positive for Alcian blue and negative for periodic acid-Schiff staining. Together with the morphological findings, no gastric-type phenotypes were observed. Other immunostaining demonstrated that CEA was negative, p16 was generally negative, and the Ki67 labeling index was less than 1%. Thus, multiple NCs and TCs were found at the 3-10 o’clock position of the uterine cervix, but no LEGH. The TCs were of the cystic type and had a maximum size of <4 mm. There appeared to be a continuity between the NCs and TCs in some areas (Figures 3A, 3B). No squamous intraepithelial lesions were observed.

The patient was under observation for a little less than one month from the surgery. However, the policy was to terminate follow-up and recommend a return visit when symptoms appeared. The patient provided informed consent for the publication of this report.

## Discussion

Two similar cases have been reported, as far as we could find, with the PubMed database. The search terms were NC, TC, or LEGH. Ando et al. reported the clinicopathological data of six cases that received hysterectomy out of 42 follow-up cases clinically suspicious of LEGH [1]. These follow-up cases tested positive for gastric-type mucin, as confirmed by a latex agglutination test using HIK1083 antibodies. Of the six pathologically confirmed cases, five were LEGH with or without atypia, and one was an NC with a TC [1]. Takatsu et al. reported a case of NCs and TCs on MRI mimicking LEGH [4]. Thus, NCs with TCs can be clinically misdiagnosed as LEGH, as observed in our case.

Ando et al. have proposed diagnostic criteria for LEGH, NC, and GAC [1]. If MRI shows a cosmotic pattern or microcystic pattern, cervical cytology shows no atypical cells, and gastric-type mucin is detected, there is a high possibility of LEGH. If MRI shows a coarse cystic pattern, cervical cytology shows no atypical cells, and no gastric-type mucin is detected, NC can be suspected. If MRI shows a solid (or microcystic) pattern and atypical cells on cervical cytology, GAC can be suspected [1]. In this case, evaluation of gastric-type mucin was not performed, but the MRI findings retrospectively favored a coarse cystic pattern rather than a typical cosmos or microcystic pattern, and there were no abnormal cells in the cervical cytology, suggesting NCs rather than LEGH.

The microcystic pattern on MRI findings may sometimes be difficult to distinguish from GAC [1]. Cases of deep NCs (and TCs) are difficult to clinically differentiate from GAC, particularly from well-differentiated GAC (previously termed minimal deviation adenocarcinoma) [8,9]. Therefore, we believe that it was necessary to histologically evaluate this case. Concerned that a punch biopsy might not lead to a diagnosis, and because the patient did not wish to preserve her fertility, we opted for a conical resection without a punch biopsy. Takatsu et al. reported that, among 54 cases of LEGH, none of the 21 cases with preoperative punch biopsy were diagnosed with LEGH. They also reported that, among 30 cases of NC and/or TC, only eight cases were diagnosed as NC and/or TC by preoperative punch biopsy [4]. The positive diagnosis rate for preoperative punch biopsy of well-differentiated GAC (previously termed minimal deviation adenocarcinoma) is reported to be 0%-62.5%, which is not high [10,11]. Thus, a large tissue sample, such as conization tissue, may be necessary for a definitive diagnosis. We used conization because the loop electrosurgical excision procedure (LEEP) method may be inadequate for evaluating cystic lesions. However, for women who wish to remain fertile, minimally invasive diagnostic procedures are necessary to avoid the disadvantages associated with conization during pregnancy. The diagnostic modalities (MRI, cervical cytology, and evaluation of gastric-type mucin) proposed by Ando et al. to differentiate LEGH, NC, and GAC may be useful for minimally invasive evaluation [1]. If the latex agglutination test cannot be used to determine gastric-type mucin, punch biopsy is likely useful for histopathologically determining gastric-type phenotypes [12-14]. LEGH is known to often differentiate into pyloric glands (gastric-type traits), whereas NCs and TCs do not usually have gastric-type traits [12].

Multiple cystic TCs are observed in the inner cervical walls. However, multiple TCs are difficult to recognize on MRI images because they are small, with a maximum histological size of less than 4 mm. Generally, TCs are found incidentally microscopically without clinical imaging recognition, are confined to the inner 1/3 of the cervical wall, are often multiple, and tend to be seen more frequently in women who are multigravid and over 30 years old [5,7,15]. The average maximum diameter of cystic TCs is less than 4 mm [5,7], which is also true in this case. Thus, the high-intensity areas identified on MRI appeared to correspond to superficial and deep NCs. The characteristic MRI findings of LEGH show two patterns: a cosmotic pattern and a microcystic pattern [1,4]. In this case, the cosmos pattern-like finding corresponded to a combination of small superficial and deep NCs, and the microcystic pattern-like findings corresponded to small superficial NCs. Therefore, it should be noted that multiple NCs, particularly including deep NCs, may be misdiagnosed as LEGH by MRI. Takatsu et al. reported a case of NCs and TCs on MRI images, in which small and large cysts were clustered together, and small cystic lesions were considered possible TC lesions [4]. In the present case, a retrospective study suggested that a slightly small cyst-like lesion could be a TC, but such a lesion would be recognized as a lower-intensity lesion rather than an NC.

NCs of the uterine cervix occur within the cervical mucosa and sometimes extend deep into the cervical stroma [16]. NCs develop during the healing process in chronic cervicitis. Specifically, in the squamocolumnar junction region, the opening of the cervical gland is closed by the proliferation of stratified squamous epithelium, resulting in the formation of NCs, which are assumed to occur near the external uterine opening (external os). However, in 175 pathologically evaluated surgical cases of Stage I uterine cancer, NCs were found in 147 cases, 73% (108 cases) of which were located near the internal os, rather than the external os [17]. This suggests that the mechanism of closure of the cervical gland opening by stratified squamous epithelium at the squamocolumnar junction appears to be minor. In the present case, most NCs were formed at a distance from the external os; there was no chronic inflammation or fibrosis in the area of the NCs, and the NCs, accompanied by TCs, focally showed continuity with TCs, suggesting that TCs might be the origin of the NCs. Segal and Hart noted in their review of 29 cases of cystic TCs that they were commonly associated with multiple NCs that occasionally penetrated deeply [5]. Takatsu et al. also reported that, among 30 cases of NC and TC, there were five cases in which both were combined [4]. NCs and TCs may represent a series of pathological conditions, and TCs may be present in the background of multiple NCs.

## Conclusions

A 48-year-old woman with multiple small and large cystic lesions in the cervix on ultrasonography was suspected to have LEGH, mainly based on MRI findings suggestive of a “cosmos” pattern and a microcystic pattern. However, pathological examination of the conization tissue revealed no evidence of LEGH. Instead, superficial and deep NCs and TCs were simultaneously identified. The high-intensity areas identified on MRI appeared to correspond to superficial and deep NCs because TCs were difficult to recognize on MRI images, as they were small. Multiple NCs might have originated from TCs. Clinicians and pathologists should be aware of the possibility of superficial and deep NCs and TCs coexisting and presenting with MRI findings that mimic LEGH. If multiple, various-sized cysts are found on the uterine cervix, NCs, LEGH, and GAC should be differentiated using MRI imaging patterns, a latex agglutination test, or punch biopsy to confirm whether the cervical mucin is gastric type, and cervical cytology to evaluate the presence of atypical cells. If fertility preservation is not necessary, a large tissue sample, such as conization, may be helpful for confirming the diagnosis.
